# How Driveability Is Conceptualised in Harness Racing: Insights from Trainer and Driver Interviews

**DOI:** 10.3390/ani16213477

**Published:** 2026-11-03

**Authors:** Lina Nyberg, Mette Pökelmann, Malin Connysson, Susanne Lundesjö Kvart, Elke Hartmann

**Affiliations:** 1 Faculty of Educational Sciences, University of Helsinki, 00014 Helsinki, Finland; lina.nyberg@helsinki.fi; 2 Mettes Horsework AB, 168 68 Bromma, Sweden; metteshorsework@gmail.com; 3 Department of Animal Biosciences, Faculty of Veterinary Medicine and Animal Science, Swedish University of Agricultural Sciences, 750 07 Uppsala, Sweden; malin.connysson@slu.se; 4 Department of Equine Studies, Faculty of Veterinary Medicine and Animal Science, Swedish University of Agricultural Sciences, 750 07 Uppsala, Sweden; susanne.lundesjo-kvart@slu.se; 5 Department of Applied Animal Science and Welfare, Faculty of Veterinary Medicine and Animal Science, Swedish University of Agricultural Sciences, 750 07 Uppsala, Sweden

**Keywords:** harness racing, training, driving, trotter, equine, horse welfare, performance

## Abstract

Within harness racing, the concept of driveability has received little scientific attention despite its apparent relevance to training, racing, and horse welfare. This study explored, through semi-structured interviews, how eight Swedish harness-racing trainers and drivers conceptualise driveability and relate it to performance, rein tension, equipment use, and horse welfare. The data were analysed with reflexive thematic analysis. Three themes were developed: (1) calm training is key, (2) one cannot always influence driveability, and (3) equipment is a tool. Participants described a driveable horse as calm, attentive, and responsive to the driver’s signals. The findings suggest that driveability is best understood as a relational and context-dependent concept, shaped by training and ongoing interaction between driver and horse, while also being influenced by individual horse characteristics, situational conditions, and equipment use. Participants described both potential alignment and tension between welfare and performance. These perceived relationships require further investigation through independent assessment of practices and welfare outcomes.

## Simple Summary

Harness racing is a global industry that faces horse welfare challenges comparable to other equestrian sports. One way of supporting horse welfare is to better understand how trainers and drivers develop effective communication with their horses, as reflected in driveability. Broadly, driveability can be understood as how easily a horse can be driven, but little research has explored how harness-racing practitioners themselves conceptualise this construct. In this study, we interviewed eight Swedish harness trainers and drivers about what they consider to be good driveability and how they think it relates to training, rein tension, equipment, performance, and horse welfare. Participants described a driveable horse as calm, attentive, and responsive to the driver’s signals. They emphasised patient training, careful adjustment to the individual horse, and thoughtful use of equipment, while recognising that a horse’s temperament and the training or racing situation also influence driveability. Together, these findings suggest that driveability is not simply a characteristic of the horse but is shaped through the ongoing interaction between driver and horse and the context in which they work together. A better understanding of driveability may help guide decisions about training and equipment choices in ways that support both performance and horse welfare.

## 1. Introduction

Harness racing, like other equestrian sports, faces ongoing questions about how horse welfare is protected in training and competition [1]. For example, mouth lesions have been reported at high prevalence in studied trotter populations [2,3,4] and certain bit types have been associated with the presence and severity of lesions [4]. In addition to bit type, excessive rein tension may be associated with oral discomfort and/or pain [5], which may be expressed through conflict behaviours such as mouth opening [6,7]. These welfare concerns are closely related to the driver–horse interface, where communication is primarily mediated through the reins, in contrast to ridden horses where riders can also influence the horse via leg and seat aids. Consequently, strategies to safeguard horse welfare are likely to benefit from an understanding of how industry professionals conceptualise optimal communication and responsiveness in the trotting horse, and how such qualities are developed in training. Within harness racing, this set of qualities may be referred to as “driveability”.

Successful preparation for harness racing requires not only physical conditioning but also habituation to equipment and racing-related environments, together with learning to respond appropriately to the driver’s signals. In Swedish harness racing, initial training commonly begins at approximately 12–18 months of age and is then gradually progressed during the following winter, with the aim of preparing horses for their first trial and qualification races. Standardbreds are allowed to race from 1 July the year they turn two, and Coldblooded trotters from the year they turn three [8]. Among stakeholders and the public, concerns have been raised about training and racing at an early age and advancing too quickly during initial training [9,10]. In this context, physical health has received greater scientific attention, and research suggests that early training and racing are associated with lower injury risk and longer racing careers [10,11,12]. Less is known, however, about how the timing and progression of initial training relate to horses’ mental states and human–horse interaction. Recent research has begun to highlight individual differences in horses’ mental maturity and their importance for informing handling and training decisions [9,13]. Equitation science further emphasises that learning is supported by clear and consistent signalling, appropriate timing of pressure and release, gradual habituation, and training conducted when the horse is in a mental state conducive to learning [14]. How these considerations relate to practitioners’ understandings of driveability remains unclear. Although driveability is a familiar concept in harness racing practice, it has received little scientific attention and lacks a shared conceptual definition.

In contrast, the related construct of “rideability” has been more widely studied in ridden horses and is an integral part of breeding evaluations and young horse performance tests [15,16,17]. Rideability broadly describes the ease and quality with which a horse performs under saddle [18,19] and is closely related to traits such as temperament and willingness to work [16]. It is typically assessed by judges and test riders through a combination of behavioural and movement-related parameters such as responsiveness to the rider’s aids and quality of rein contact. Attempts to identify objective indicators of rideability have produced mixed findings. While behavioural indicators such as conflict behaviours have been associated with lower rideability scores [18,19], studies relating rein tension to rideability have reported inconsistent results [19]. This may partly explain the remaining subjective component of rideability scoring, as riders may relate more to the perceived quality of rein contact rather than the quantity of rein tension per se [15,19]. Likewise, in a harness-racing context, Hartmann et al. [20] found that measured rein tension was not associated with drivers’ ratings of driveability during race training, although horses estimated by drivers to have heavier rein tension tended to receive higher driveability scores. Overall, existing research illustrates the complexity of assessing human–horse communication and responsiveness to signals. How these qualities are conceptualised within harness racing, however, remains largely unexplored.

In ridden-horse contexts, breeding organisations in several European countries have attempted to formalise the assessment of rideability and related temperamental traits through structured evaluation protocols. For example, within the Swedish Warmblood breeding system, rideability is assessed through temperament and general impression scores when horses are evaluated for gaits under the rider [21]. Rideability is also formally assessed in standardised breeding field tests in Icelandic horses via the International Federation of Icelandic Horse Associations (FEIF), where emphasis is placed on cooperation, responsiveness, relaxation, alertness, and willingness to perform. Evaluation rules further specify that the horse should remain light on the reins, mentally balanced, and perform with minimal aid from the rider [22].

Although racing performance remains the primary breeding objective in trotters, several breeding programmes also include behavioural and temperament-related traits relevant to driveability [23,24,25]. For Swedish Standardbreds, these traits are addressed indirectly through breeding objectives such as ease of handling, rather than through a standardised assessment explicitly evaluating driveability or temperament traits [23]. For evaluations of Swedish Coldblooded trotter stallions, a voluntary driving test may be included as part of the breeding evaluation, assessing primarily gait characteristics and pulling aptitude but also temperament and ease of handling [23]. In Finland, driving tests are also used in breeding evaluations, although their requirements differ between breeds. For Finnish Coldblooded trotter stallions, a driving test assessing temperament, including cooperation and willingness to move forward, as well as trot biomechanics, is mandatory, while it is optional for mares evaluated on the basis of their progeny [25]. The Finnish Standardbred breeding programme likewise includes a formal driving test in which traits such as alertness, flexibility, calmness, and cooperation are assessed while the horse is driven at a walk and trot, although the test is optional for stallions and mares evaluated on the basis of their progeny [24]. Recent work suggests that temperament dimensions assessed in Standardbreds, such as “agreeableness” (including cooperation and willingness) are positively associated with racing performance, e.g., times and earnings [26]. Such traits may capture aspects of functional driveability, as horses must be sufficiently driveable to qualify, start, and compete successfully.

Taken together, these breeding objectives and assessments indicate that behavioural traits relevant to driveability are already recognised within harness racing, although they are described and evaluated in different ways. However, the broader concept of driveability itself remains poorly defined, and little is known about how experienced trainers and drivers conceptualise it in everyday practice. Drivers and trainers possess extensive practice-based knowledge of driveability, making semi-structured interviews a suitable approach for exploring how the term is understood in practice. A clearer understanding of how stakeholders conceptualise driveability may help identify the qualities they value in driver–horse communication and the reasoning behind training and equipment choices, providing a basis for examining how these priorities relate to horse welfare. The aim of the current study was to explore the concept of driveability from the perspective of harness-racing trainers and drivers. The overarching research question was as follows: How do Swedish harness-racing trainers and drivers conceptualise driveability, and how do they relate it to performance, rein tension, equipment use, and horse welfare?

## 2. Materials and Methods

This study used a qualitative design based on semi-structured interviews with Swedish harness-racing trainers and drivers to explore the concept of driveability and capture participants’ experiential and practice-based knowledge.

### 2.1. Ethical Statement

According to the Swedish Ethical Review Act [27], ethical approval was not required because this study did not involve (i) processing of “sensitive” personal data, (ii) physical interventions, or (iii) methods intended to affect participants physically or mentally. Interviews were conducted with adult participants regarding their professional experiences, and all data were pseudonymised prior to analysis and reporting (i.e., identifying details were removed and participants are referred to by pseudonyms). Because gender was not a variable of interest, all participants were assigned gender-neutral pseudonyms.

All interview participants were fully informed about the study’s purpose, the main contact person for the study, and their right to withdraw at any time. Participation was voluntary, and written informed consent, including consent to audio recording, was obtained prior to interviewing.

### 2.2. Participants

Interview participants were recruited using purposive sampling combined with an element of random selection. Recruitment considered the following factors: A- or B-trainer licence status, minimum age of 18 years, having experience in training and racing both Standardbred and Coldblooded trotters, and variation in gender and geographical regions. Based on these criteria, an initial sampling list was compiled from the top 200 trainers and drivers obtained from the 2023 Swedish harness-racing statistics, ranked by the number of wins within their respective A or B-trainer licence category. This list was screened against the inclusion criteria, and 20 eligible practitioners were contacted individually in random order by email and/or telephone.

In total, eight trainers and drivers agreed to participate, while the remaining 12 practitioners did not respond to the invitation. Eight interviews were considered sufficient for this exploratory analysis because participants had relevant experience and provided detailed accounts supporting the development of the three themes.

The interviews were conducted between November 2023 and May 2024. Four participants held an A-licence (professional trainer licence) and four held a B-licence (training is limited to horses for which the holder is the legal representative/authorised signatory, plus a maximum of five additional horses). Four interviewees were female and four were male, and they were aged between 26 and 62 years (median age of 49.5 years). They represented five racetracks across Sweden and had a median of 75 official race starts as trainers (range: 0–228) and a median of 119 as drivers (range: 0–572) according to official 2023 racing statistics.

### 2.3. Theoretical Framework for Interview Questions

The interview questions were developed within an animal welfare science framework, specifically the five domains model [28], recognising that welfare in equine sport emerges from the interplay between the horse’s physical state, management-related conditions, and the quality of human–horse interactions that, together, influence horses’ affective (subjective) experiences. The interview questions were also informed by an equitation science framework [14], which emphasises that welfare-relevant outcomes emerge from how signals are applied, perceived, and learned by the horse. In addition, welfare discussions in harness racing increasingly focus on the use and regulation of driving equipment (including bits) and on the potential welfare consequences of sustained or high rein tension [2,4,5], making these topics important to explore alongside stakeholders’ conceptualisations of optimal driveability. Together, these considerations informed the development of interview questions relating to: (1) optimal driveability (stakeholders’ understanding of effective communication and cooperation with the horse), (2) rein tension and equipment choices (key interfaces of communication and potential welfare risk), and (3) horse welfare considerations (how participants connect training and racing practices to horse welfare). Finally, we included questions on (4) knowledge-seeking behaviours and the potential value of objective measurement tools for providing feedback on both performance and welfare, as previously explored in thoroughbred racing [29].

### 2.4. Interview Procedure

The interview guide was piloted by the interviewer with two harness-racing stakeholders who were not included in the final sample. Minor refinements to the wording and order of questions were made to improve clarity and flow.

All interviews were conducted in Swedish by the second author, who has experience of harness racing but no prior personal or professional relationship with any of the participants. This experience facilitated contextual understanding and rapport with participants. Interviews followed a semi-structured guide focusing on the four topic areas outlined above. While the wording and order of questions varied slightly depending on the course of the conversation, all participants were asked about the same topics. Interviews were held face-to-face on racetracks or farms and lasted approximately 60 min. They were audio-recorded using a digital recorder and transcribed verbatim in Microsoft Word. Transcripts were checked against the audio recordings by the interviewer to ensure accuracy.

### 2.5. Data Analysis

The data were analysed using reflexive thematic analysis as described by Braun and Clarke [30,31]. The first author, who conducted all stages of the analysis, including coding, has a background in educational sciences and extensive experience with horses through practice, teaching, and research. This background informed the interpretation of participants’ accounts while also requiring ongoing reflexivity regarding assumptions about equine training and welfare.

Coding was conducted manually; Microsoft Word was used to annotate transcripts and organise codes and candidate themes. The last author read the transcripts to support discussion of the developing interpretations but did not independently code the data. The fourth author contributed to analytical discussions when questions arose and helped refine the presentation of the findings.

Following the six-phase process described by Braun and Clarke [30], the analysis began with familiarisation with the data. All interview transcripts were read twice, and all interviews were listened to twice, each time in different orders. As researcher reflexivity is an important aspect of reflexive thematic analysis, brief notes were made in this familiarisation phase regarding overall impressions of the data. Following familiarisation, the entire data set was coded systematically by hand, capturing primarily semantic meaning from the data segments. After initial coding, the data set was re-read, and codes were reviewed and modified three times before moving on to developing potential themes. This phase of the analysis was a circular process, as some codes were refined to better capture meaning or discarded as not relevant to the research question. Codes were clustered or collapsed into larger entities of meaning and potential themes were then generated from these, keeping the main research question in mind, i.e., how stakeholders conceptualise driveability and how they relate it to performance, rein tension, equipment use, and horse welfare.

As themes were developed and refined, the analysis also became more interpretive and focused on identifying underlying patterns of meaning and relationships between participants’ accounts. The process remained primarily inductive while being informed by the study’s research question and theoretical perspectives. Initially, five themes were generated, and preliminary theme descriptions were written to assess the depth, central organising concepts, and relevance of each theme to the research question. As some themes lacked depth or overlapped conceptually with others, the codes and related data segments were re-assessed and re-organised into three final themes, which were also evaluated for depth, central concept, and relevance. At this stage, the transcripts were re-read to ensure that the analysis remained grounded in the data. Finally, the themes were refined into a coherent narrative of participants’ conceptualisations of driveability, and illustrative data segments were selected to support the interpretation. These quotations were translated into English with the assistance of generative AI in the later stages of manuscript editing. All translations were subsequently reviewed and, where necessary, revised by the last author, who is fluent in both Swedish and English, to ensure accuracy and preserve the meaning and tone of participants’ accounts. The analysis was informed by a critical realist perspective, a philosophical approach that combines a realist ontology with a critical epistemology [32,33]. Within critical realism, reality is accepted to exist independently of human perception, while human knowledge is regarded as always mediated by social, cultural, and historical factors. Accordingly, interview accounts were understood as participants’ interpretations of real-world experiences rather than direct representations of reality or evidence of practices and their direct outcomes. Participants’ conceptualisations of driveability were therefore interpreted as experience-based understandings of phenomena encountered in practice, while recognising that these understandings were shaped by the social, cultural, and professional context of Swedish harness racing.

## 3. Results and Discussion

Three themes were developed that address how participants conceptualised drivea Participants’ shared descriptions of a driveable horse are first presented as an analytic synthesis, providing an important point for understanding the subsequent thematic findings (Table 1).

### 3.1. Conceptualisation of Driveability

When discussing driveability, participants were highly consistent in their responses: a horse with optimal driveability was described as calm and relaxed yet alert, listening and responding promptly to the driver’s signals to accelerate, decelerate, and turn.

*Elia: The starting point is that they should respond to aids, and that you should be able to manage both their speed and steering. That is really what you want—they should simply do what we want them to do. If we want them to go fast, they should do so; if we want to slow them down, we should be able to do that. If we turn right, they should turn right, and that’s basically it.*

*Bobbie: A horse that is relaxed and responds immediately to what you ask of it.*

Participants’ understanding of driveability overlaps with key elements often discussed under “rideability” in ridden horses, particularly calmness, attentiveness, and responsiveness to human signals [15]. An important finding was the high degree of conceptual consensus among participants, particularly the emphasis they placed on calmness. This emphasis provides an entry point to the themes below, where calmness is described as both trainable and welfare-relevant, and as important for performance under racing conditions.

### 3.2. Calm Training Is Key

This theme highlights calm training as key to driveability, performance, and horse welfare. Participants described calm training through gradual progression and low-intensity training as an approach to developing responsiveness. This was understood to facilitate driver–horse communication, support performance, and help negotiate the welfare challenges associated with competitive racing.

Training situations that improved driveability were often described by participants as calm in their entirety. Hence, calm training was described as a broader approach extending across training, racing and recovery decisions. Participants referred primarily to the horse’s mental state alongside physical aspects, such as relaxed posture and movement. Across the data, central components of this approach included low-intensity training and careful initial training of the young horse.

*Bobbie: I think it helps a lot if you walk the horses before and after training sessions. Instead of rushing out of the stable at full speed to the training track and then rushing home again at full speed. The more calm periods you can have driving the horse, the better. And that may mean going out one day just to jog quietly for 4, 5, 6, or 7 kilometres, even when you are not really supposed to train.*

*Mika: You can of course train that [driving young horses] very well too, but there are no shortcuts where you can skip a couple of steps with young horses and think it will turn out well. It may work with individual horses that are very calm and easy, but not all horses will be okay with that.*

Bobbie’s and Mika’s comments illustrate how calmness is achieved through an unhurried approach throughout the horse’s training. Participants emphasised the importance of starting early, without rushing, and of stepping back when necessary. In line with a previous study on racing stakeholders’ perspectives [9], participants in the present study did not frame early training as problematic in itself; rather, their accounts centred on the avoidance of rushed or forced approaches. This distinction is relevant because the appropriate age at which to begin training and competition remains debated [9,10,12,13].

The emphasis on calmness extended beyond early training, where adapting the type and intensity of training to the individual horse was described as a way of maintaining calmness. Some participants’ accounts suggest that both accumulated experience and the absence of external pressure may enable such approaches, as illustrated by Elia:

*Elia: But you also step back when training horses if you feel that things are starting to get out of hand and have gone in the wrong direction and give them the time they need. Since I do this on an amateur basis, I do not have to push because of some horse owner who thinks, ‘now damn it, we are going to start here,’ but I can be a bit more relaxed—if they start in the autumn as three-year-olds, I am happy with that. They don’t have to be out racing during the year they are three.*

Calm training, as characterised by participants, can thus be understood as an approach that is responsive to the horse’s behavioural signs and individual needs. Across the data, participants described training practices that may be interpreted in relation to key learning principles such as habituation and gradual exposure. This was reflected particularly strongly in their emphasis on developing and maintaining a calm state in the horse, although this was expressed through descriptions of practice rather than learning-theory terminology. Notably, participants did not describe in concrete terms how they teach horses to respond to signals. Instead, they emphasised that their overall training approach—rather than specific techniques for teaching such responses—contributes to horses becoming driveable:

*Chris: You can train with a lot of low-intensity, calm sessions, for example walking a lot and jogging a lot and often. And then perhaps gradually move on to more regular training, but that too can be organised in a very calm way. It may train at low speeds and do long intervals and not so many short intervals. Yes, I think it is mainly through the training that you can educate the horse, and it is probably difficult to educate it in other ways.*

While participants’ training practices appear consistent with key learning principles, the absence of explicit descriptions of how horses were taught to respond to signals suggests that these processes may not always be conceptualised or articulated in learning-theory terms. Previous research indicates that equine professionals may have limited explicit engagement with learning theory terminology, even where practical understanding is present [34,35,36]. Such knowledge may remain tacit [37,38]. However, the interviews cannot determine participants’ actual knowledge or application of learning theory in practice.

This distinction between practical knowledge and explicit learning-theory concepts was also reflected in participants’ accounts of how they addressed driveability problems, such as excessive rein tension when slowing down, the horse “pulling hard” (on the reins), or being described as “hot”. By hot, participants referred to a horse that is agitated, stressed, and takes excessive rein tension—an undesirable, hard-to-control condition that they sought to prevent through calm, low-intensity training routines. Driveability issues were commonly attributed by participants to horses having been pressured or trained too intensively. Participants described a range of strategies to improve driveability, many of which involved adjusting routines—such as training approach, warm-up, and racing decisions—to help the horse regain calmness:

*Kay: You try to avoid getting them too fired up, and that includes everything from the training set-up, to the warm-up, to the competition itself, and even a little how you enter them in races.*

None of the respondents explicitly referred to training the horse to maintain lighter contact or to respond to more subtle rein signals. Thus, although such principles may have been applied in practice, the interviews provide no evidence that participants consciously used or articulated them as strategies for improving driveability. From an equitation science perspective, negative reinforcement relies on timely and consistent pressure–release, with training aiming to achieve responses from the lightest effective signals [14]. Given the high rein tensions reported in harness racing [5,20,39], this warrants further investigation. High or inconsistent rein tension may be problematic if pressure and release are not applied clearly and consistently. Such inconsistencies have been associated with conflict behaviours [6,40], suggesting that they may be relevant to some of the driveability problems described by participants (e.g., “pulling hard,” difficulties slowing down, being “hot”). In this context, reduced driveability may also raise safety concerns, as diminished control has implications for the safety of both horse and driver. This is consistent with other work showing that undesired behaviours matter for horse welfare and human safety [34,41,42]. Although participants did not explicitly frame driveability problems as safety issues, their descriptions of highly aroused or “hot” horses highlighted difficulties in maintaining control, with potential consequences for both horse and driver. Robin’s account illustrates how such situations prompted a deliberate shift in training approach:

*Robin: So it got to the point where this was going to be the last attempt, to try doing something completely different with him and see if we could get him to calm down a bit. So, I do not drive him fast at all; instead, we do everything else. We go out walking a lot, he is ridden, he is long-reined, and of course I also have to drive him a lot for exercise. He is still energetic, but it is much better. It is not like I feel I have absolutely no control over him. He has also raced twice for me now, and of course you can still see that he is energetic, but touch wood, it does not look unmanageable.*

Robin’s account illustrates how challenges associated with a highly aroused horse prompted an increased emphasis on calm, low-intensity work. The revised training approach was described as improving the horse’s manageability and restoring the driver’s sense of control.

Participants also described calm training as supporting performance under racing conditions, as a calm horse was considered better able to attend to and respond to the driver’s signals. Some participants referred to this as cooperation, and others to obedience, but in both cases, the desired outcome was the same: prompt responses to signals to regulate speed and direction during the race. According to participants, this was particularly important during the start phase of the race, where responsiveness and control are required to avoid a poor start and reduce errors that may lead to penalties or disqualification, such as breaking into gallop, as Mika points out:

*Mika: […] We have, for example, a volt-start system, and there the horse needs to be in balance in order to trot cleanly when turning up into the start and circling. It is similar behind the starting car. For example, with horses that are a little too eager, and that for some reason you do not want to send off too fast, or that cannot start that fast, they need to accept being steadied and allowing a few other horses to pass them at the start because you want to take it calmly. And this is a great advantage if they can cope with that, because then you avoid a lot of galloping errors.*

Participants also discussed horses’ use of energy in relation to driveability and performance. They described calm training as helping to prevent horses from becoming “hot” and, in their view, using energy unnecessarily before or during the race. Chris further suggested that this could affect immediate race performance and the horse’s ability to perform consistently across repeated competitions:

*Chris: Of course, if you have a horse that is too ambitious and does not listen to its driver, it can overexert itself both before and during the race, at times when it is not really supposed to work that hard, and that can of course leave it depleted of energy. … But I think the main problem is when the horse becomes too stressed and agitated. Some horses can cope with that, but of course they should not be using up part of their energy, and they may not be able to race as often because they are performing at maximum effort. Then they really empty themselves completely, because they have been stressed and exerting themselves before the race and for a long time during the race, instead of just doing their job and listening to the driver.*

Importantly, participants did not attribute energy use solely to physical work. Instead, they repeatedly linked it to horses becoming stressed or excessively aroused before or during racing. Although driveability has received little direct attention in relation to racing performance, this interpretation is consistent with previous research showing that higher pre-race arousal and stress are associated with poorer performance [43,44,45].

Finally, participants discussed calm training in relation to what they perceived as good horse welfare. As described above, participants regarded calmness as central to driveability and sought to develop it through unhurried approaches and by remaining responsive to the horse’s behaviour and readiness to learn. Across the interviews, these practices were consistently described as beneficial for the horse and are interpreted here as reflecting concern for the horse’s welfare. Some participants explicitly connected driveability to wellbeing:

*Billie: Of course, if the horse is very responsive, easy to drive, and going well, then it is almost guaranteed that it is also feeling well and is content with its life and with what it is being asked to do at that moment […]*

However, responsiveness and ease of driving cannot, in themselves, establish whether a horse is experiencing positive welfare. Contemporary welfare frameworks go beyond these characteristics alone [28]. Within the scope of the interviews and participants’ roles as trainers and/or drivers, horse welfare was nevertheless discussed in terms of physical health, mental state, and human–horse interactions. In particular, welfare was described by participants in relation to horses’ emotions and experiences during training and racing. Calm training was presented as contributing to what participants interpreted as positive experiences, motivation, and willingness to engage, while fatigue was described not only as a physical strain but also as influencing the horse’s experience of the situation. As illustrated by Jessie and Mika, horses were expected to “have fun” and “feel capable”:

*Jessie: I don’t want them to come back with that lactic-acid taste in their mouth; I want them to feel that it was enjoyable. And they shouldn’t come back to the stable completely out of breath. They should come back feeling that it was nice to be out driving. They mustn’t be completely exhausted.*

*Mika: You could say that we always finish with a much calmer interval. I mainly do that because, as we discussed earlier, I don’t want them to leave the track completely exhausted. Instead, we do one more heat, but never above a heart rate of 200 in the final heat. It may be at only a 45-50 pace, so they can jog down a little while still moving at some speed. Then most horses feel, ‘I can manage this just fine.’ And then they leave the track feeling okay, which is what you want.*

These accounts reflect an interest in the horse’s subjective experience that is consistent with recent research suggesting a shift towards a more holistic understanding of welfare among equine stakeholders, including greater attention to horses’ mental experiences and emotions [46], and with broader calls to recognise the horse’s subjective experience as central to welfare [1,47].

Participants nevertheless recognised that maintaining horse welfare was not straightforward within competitive racing. Racing was described as inherently demanding, requiring horses to perform at a high level in environments that were not always considered optimal from a welfare perspective. Factors such as travel, unfamiliar surroundings, and exposure to loud noise were identified as sources of stress. Previous studies have similarly shown that horses exhibit physiological stress responses associated with transport and competition [45,48]. Beyond these immediate demands, participants also reflected on broader time constraints within racing. The relatively short competitive careers of trotters meant that participants sometimes had to balance what was considered ideal for the horse with what was feasible within the competitive context:

*Kay: We have a racing career that can vary in length. There are certain things you have to deal with, otherwise it simply does not work, while there are other things you can more or less live with. That applies to everything from bits and pressure to the horse as a whole. If you knew you had five or six years with every horse, you might do some things a little differently.*

Kay’s account illustrates how participants described balancing welfare considerations with the practical constraints of racing, including time pressures and performance expectations. This tension between welfare aspirations and the realities of competitive horse sport has also been recognised in broader discussions of equestrian welfare and governance [1,49].

Against this background, participants described calm training as a way of negotiating these welfare challenges. They regarded calmness as central to driveability and described calm training as helping horses remain calm and responsive despite the demands of racing. This interpretation is consistent with previous research suggesting that welfare-related impacts may be mitigated through appropriate management and training [50]. Although participants primarily emphasised the benefits of calm training for the horse’s mental state, some also suggested that progressing gradually and training in a calm manner could reduce the need for veterinary interventions and help maintain physical health:

*Elia: I use the veterinarian much less nowadays than I used to, and that is because I feel I can train at a level where we no longer have to push beyond the limit. Then things still happen where you need help from a veterinarian, but for a while there was this mentality of just ‘inject and race’, that is not my thing. It did not really feel like me. We have spent an enormous amount of money on veterinary treatment over the years and had incredibly poor results. So I know that is not the answer.*

The expression “inject and race” here appears to refer to treating the horse’s joints or tendons to avoid interrupting competition. While such treatments may be used to alleviate pain and support welfare, they may also enable continued racing despite underlying issues [49]. Elia’s account reflects both ethical discomfort and dissatisfaction with this approach, which, in this participant’s experience, was associated with poor results despite substantial veterinary treatment.

Beyond these practical considerations, some participants also reflected on the broader ethical responsibilities associated with using horses in competitive sport. Such reflections were mostly implicit and framed in terms of providing horses with a good life outside competition, fostering positive human–horse relationships, meeting the horse’s basic needs, treating the horse “fairly”, and preparing it appropriately for racing through calm, gradual training. As reflected in Billie’s account:

*Billie: Yes, what we do is a strange sport, because really it is difficult to justify any of it. We think it is acceptable to, more or less, force horses to run round and round against the clock in order to win money if they are the best at it. But having said that, because it is in fact accepted to do this and […] with the way I personally do it, I still think it is okay […] because you are very careful about absolutely everything concerning the horse and how it is doing […]*

These accounts suggest that participants engaged in nuanced welfare reasoning that, for some, extended to explicit ethical reflection on the justification of using horses in competitive sport. Such ethical reflection has previously been proposed as an important component of responsible equestrian practice [51].

Taken together, this theme highlights calm training as central to participants’ understanding of driveability, performance, and welfare. The analysis suggests that participants viewed these as closely interconnected, and perceived calm training as beneficial to all three.

### 3.3. One Cannot Always Influence Driveability

The second theme centres on participants’ accounts of driveability as something that cannot always be influenced, as it is also shaped by the horse’s inherited temperament, biomechanics, and situational factors beyond the driver’s control. In this section, we present participants’ accounts of how these factors were understood to influence driveability and how they adapted their strategies when driveability could not be readily changed.

Across interviews, participants repeatedly described “bloodlines”, “mentality”, and “temperament” as shaping how easily a horse can be driven, and some suggested they could often predict driveability challenges based on pedigree. These challenges could be “hotness”, excessive sensitivity, or “hardness”, as Robin mentioned:

*Robin: From experience, if you think about breeding, with some horses you can almost predict what they will be like as soon as you sit on them or get behind them, simply from their pedigree. Some lines really do produce horses that are very hard, or whatever you want to call it.*

By “hard”, Robin appears to refer to horses that are more difficult to regulate, potentially including horses that take a stronger rein contact and respond less readily to the driver’s signals. Temperament was thus associated with responsiveness to rein signals and, more broadly, with the ease of driving and handling, indicating that participants did not view driveability as determined by training alone. This interpretation aligns with previous research suggesting that traits related to driveability, such as agreeableness and excitability, show moderate heritability [26]. Agreeableness encompasses traits such as cooperation and willingness to work, while excitability may correspond to what participants described as “hotness”.

Although participants acknowledged the existence of bloodlines associated with poorer driveability, some pointed out that driveability in trotters has generally improved through breeding. As reflected in Bobbie’s account, they considered it important to take driveability into account in breeding, because they regarded a calm and harmonious temperament as influencing how easy the horse is to handle, train, and compete with:

*Bobbie: There is a big difference in the horses now compared with 15 years ago. I don’t know whether the right term is driveability, but there is a very big difference in how the horses are to handle. That applies from the time you start them in harness through to racing them. They are almost a different breed now compared with just 15 years ago, and if you went back 30 or 40 years, it would be like two different breeds. The horses used to be much, much more forward-going. Nowadays, overall, most are quite pleasant to drive, and it is extremely rare to have a horse that pulls hard in a race. But when it comes to breeding, you would not breed a hot horse to another hot horse.*

The practical importance of temperament is reflected in breeding programmes, where it is often included as a selection criterion [23,24,25]. For example, the Swedish breeding programmes for Standardbred and Coldblooded trotters include traits such as ease of handling [23], and the Finnish equivalents include traits such as calmness and cooperation, which may be assessed in relation to the horse’s behaviour while being driven [24,25]. Some participants, however, described driveability as a by-product of selection for racing performance, arguing that horses selected for breeding on the basis of their racing performance often also display favourable driveability and handling traits. This may reflect indirect selection, since horses with poor driveability may be less likely to achieve consistent racing performance and are therefore less likely to be chosen for breeding.

While breeding for performance may favour horses that are highly motivated to go forward, participants also described a tension: the same “keenness” that supports racing success can challenge driveability if the horse becomes too hot or hard. Racing is about finishing first, and horses are expected to be willing to compete while at the same time remaining responsive to the driver:

*Kay: We want them to be willing to go forward and compete, but at the same time we want them to listen, and that becomes a bit of a balancing act.*

*Billie: Of course, you want the horse to be responsive, because this is harness racing and, as I said, they need to be responsive. But they also need to have that explosiveness, and it is difficult to combine being completely harmonious with still being alert and ready to challenge the other horses in a millisecond. But somehow, that is what you have to strive for.*

These accounts suggest that traits associated with driveability were not always perceived as fully compatible with traits associated with competitive performance, highlighting the need to balance different characteristics valued in racing horses. As one participant expressed, a horse may need both calmness and “explosiveness”. In this sense, a more reactive or fiery temperament may, in some cases, be perceived as beneficial for performance even when it compromises driveability.

Besides temperament, participants also described other factors influencing driveability that were partly out of the driver’s control. Biomechanics—the way the horse moves—was described by some participants as influencing driveability. As Chris explained, locomotor asymmetry and compensatory movement patterns may prevent the horse from moving comfortably:

*Chris: That* [the horse hanging on one rein] *can also very often be a space-related problem, where the horse simply does not have enough room for all its legs while trotting and then it may start moving crookedly or compensating to make room, because it is uncomfortable if it strikes itself. I think that is very common.*

Several participants described “hanging on one rein”—that is, taking greater tension on one rein than the other—as a common driveability problem. Chris suggested that this behaviour may reflect underlying biomechanical limitations or discomfort. This interpretation is consistent with previous work indicating that horses with hindlimb asymmetry exhibited higher rein tensions than symmetrical horses, although the finding was based on a small sample [39].

Participants also noted situational factors that may affect driveability, including sudden events and equipment issues:

*Jessie: The mouth, I mean—equipment. Sometimes a horse may have a bit that it likes to lean on and pull against, or it may have a bit that it does not like and then pulls. There can be so many reasons: a bad day, bad weather, a deer in the forest, and so on.*

By “pulling”, Jessie refers to the horse leaning against the bit or taking a strong contact, which participants regarded as a driveability problem. Across the data, participants described behaviours such as “pulling” or “hanging” on one rein as arising from multiple interacting factors, including reactions to equipment and unpredictable environmental stimuli (e.g., wildlife or weather). In the equitation science literature, behaviours such as pulling or hanging on one rein are considered potential indicators of conflict, warranting investigation of underlying causes [40,42,52]. However, participants emphasised that the causes were not always clear or immediately modifiable during training or racing.

When driveability was reduced and participants felt unable to influence it directly, they described driving the horse in cover; that is, positioning the horse behind another horse during training or racing. The horse in front was described as acting as a “brake”, helping to reduce speed and arousal. At an appropriate moment, the driver could then steer out to pass when the horse was considered better able to respond after a period of relaxation. Participants also described this strategy as reducing the need for strong rein pressure, which they regarded as undesirable. As illustrated by Elia:

*Elia: And of course, from experience I know that if you have a horse that is a bit difficult, mentally, and that seems to run in the bloodline—that it is a bit hot—then I always drive those horses behind another horse. You try to avoid having to pull on the horse’s mouth. There is nothing worse than seeing someone hanging behind a horse and pulling. Then you know you are not cooperating. So, you are constantly trying to find ways forward where you do not really have to resort to force. And there have been horses in training that I have only driven behind another horse because they simply were not enjoyable to drive.*

Elia’s account illustrates how participants adapted their driving strategies to the horse’s characteristics and current state, seeking to maintain control without resorting to force.

Overall, this theme highlights that driveability is understood as being shaped by inherited temperament, movement patterns, and situational conditions that may limit the driver’s ability to influence it directly. Participants described adapting their strategies to manage these influences when the causes of reduced driveability could not be readily changed.

### 3.4. Equipment Is a Tool

The third theme highlights how participants understood equipment—primarily bits and reins—as a tool for achieving driveability and performance. Participants most often referred to *rein tension*. In the analysis, however, the broader term *rein contact* is used where appropriate, as participants’ accounts extended beyond rein tension itself to include how communication through the reins was experienced and interpreted. Below, we present how participants described equipment and rein contact as facilitating communication, supporting performance, and providing information about the horse, while requiring ongoing negotiation between functionality and horse welfare.

In discussing equipment, participants focused mainly on bits and reins, which was expected as these constitute the primary means of communicating direction and speed to the horse. Participants emphasised that while the horse may be walked on a loose rein, some degree of rein tension should be maintained during training and racing, but the level must be appropriate. Both excessively strong and overly light rein tension were considered problematic. Horses described as “heavy” or “too sensitive” were more difficult to communicate with, making fine-tuning of signals more challenging or increasing the risk of errors, as Mika’s account illustrates:

*Mika: Yes, if you take it to the extremes, you could say that a horse that is too heavy in the contact, that is, takes too much hold of the bit, that is not good. At the other extreme, a horse that is overly sensitive and tolerates very little contact is not good for a trotter either, because such horses are more likely to break into gallop if you take a little too much hold of them, so to speak.*

Appropriate rein contact was thus described as central to communication between driver and horse. Although an appropriate level of rein tension remains difficult to define, research suggests that horses may experience discomfort at lower rein tensions than those commonly reported during ridden work [52,53]. In race-trained trotters, considerably higher rein tensions have been reported than in ridden horses [5,39], and higher estimated rein tensions have also been associated with drivers’ ratings of increased driveability during race training [20]. These findings suggest that relatively high rein tensions may be common in harness racing. Participants similarly recognised that heavy rein contact frequently occurs in practice, although they did not necessarily regard it as desirable:

*Billie: I really don’t like fighting with them—of course you don’t—but I simply try to pick at or pull on their mouths as little as possible. Every time you drive someone else’s horse that takes a very strong hold before a race or something like that, you almost get annoyed because you feel they have done a poor job. And there is always such a bad explanation waiting if you comment on it: ‘That’s just how he is, you know.’ It is impossible that half of all horses are simply a certain way.*

*Jessie: But then, a lot of it comes down to personal preference. I want it to feel like I have a packet of butter in my hands when I drive, whereas someone else might want to have 30 kg in their hands.*

Together, these accounts illustrate that participants regarded rein contact as shaped by both driver preferences and the horse’s responses. These comments highlight the driver’s role in determining the level of rein tension applied. At the same time, participants also referred to how the horse “takes” the bit; that is, the level of contact the horse seeks or accepts. Rather than attributing it solely to either the horse or the driver, rein tension can be understood as co-produced. This aligns with previous research suggesting that rein tension reflects both the horse’s movement and behaviour and the driver’s actions [39,54].

Beyond the level of rein tension applied, participants also emphasised the importance of equipment choice for both communication and horse welfare. Kay highlighted that preventing oral injuries was a fundamental responsibility of the trainer:

*Kay: That also applies to oral hygiene, or whatever you want to call it. It is very important that you make sure you do not put anything on the horse that causes a lesion. That is absolutely fundamental, but it is our responsibility as trainers and something we have to take care of.*

Participants often attributed oral discomfort and oral lesions to driver technique, inappropriate bit choice, or prolonged use of the same equipment. They described strategies to avoid or mitigate such risks, including selecting appropriately sized and shaped bits and varying the equipment used to avoid repeated pressure on the same oral areas. Participants’ concerns are consistent with previous research showing that excessive or poorly managed rein tension may be associated with oral discomfort and lesions [5]. These accounts further suggest an awareness of potentially harmful practices, but should not be taken as evidence that such risks are absent or effectively managed in practice. Bit-related oral lesions have been reported at high prevalence in trotters [3] and broader research suggests that welfare concerns may be downplayed in equestrian contexts [55].

Within this context, choice of equipment—particularly the bit—was described as central to managing both communication and potential harm. Across the interview data, “simple” and “mild” equipment that suits the individual horse both physically and mentally was described as the starting point for equipment choice, as illustrated by Bobbie:

*Bobbie: The starting point is that the horse should have as mild and as little equipment as possible. You could say that is the first goal. […] The behaviour you are constantly aiming for is for the horse to move as efficiently as possible.*

Participants emphasised that equipment should both be comfortable for the horse and function effectively for communication and control. In participants’ accounts, these aims often appeared interconnected, as comfortable equipment was described as facilitating calmness, a more accepting rein contact, and reduced energy use by minimising attempts to evade discomfort. As Chris explained, equipment that caused irritation or unnecessary stress was considered undesirable:

*Chris: You do not want an unnecessarily stressed horse, even if it can learn if you use a piece of equipment consistently. But of course, the horse has to be comfortable with its equipment. You would not choose a bit that it seems irritated by and think that it will learn; then it is better to choose another one.*

Participants’ emphasis on both functionality and comfort aligns with previous research suggesting that perceived control is a primary motivator for riders’ equipment choices [56,57]. However, unlike respondents in Hockenhull and Creighton [56], where only 3% chose equipment because it was perceived as more comfortable for the horse, participants in the present study explicitly discussed horse comfort and potential harm in relation to equipment use. Whilst comfort and functionality were often described as closely interconnected, some participants also described situations in which short-term functionality or performance was prioritised over long-term comfort. Equipment considered beneficial for communication or performance in the short term was sometimes avoided during routine training because of concerns about its long-term effects on the horse, as illustrated by Mika:

*Mika: […] then there can be horses that go with so-called Mullen mouth regulator bits. They are not fastened differently, but we use them in races to help horses to travel a little straighter. We never use them in training, because I think that, over time, horses become, so to speak, less sensitive in the mouth if they run and train with such bits. You always have to use a little too much force with them.*

Thus, equipment choice was described as involving ongoing negotiation between functionality, performance, and horse comfort. Participants’ accounts of equipment choice when facing difficulties with driveability and/or performance further illustrate how these sometimes competing aims were negotiated in practice. Both relatively milder and sharper equipment was described as appropriate for achieving better driveability. Here, “mild” and “sharp” reflect participants’ own assessments rather than classifications established by the authors or supported by scientific evidence. On the one hand, some participants explained that milder equipment functions better, as problems may stem from pain or discomfort:

*Elia: And for me, there is no truth in the idea that a sharper bit makes them lean less […]. I have a very good example from when I was a trainer and was going to drive a trial start, but the horse was so wild and impossible to steer. In the end, the bit broke. Then the horse became steerable. So violence should not be driven out with violence.*

On the other hand, some described additional tools or sharper bits as improving driveability and reducing the need for excessive rein tension:

*Robin: […] he gets very hot, and I do not want to just sit there pulling on him, so with him, for example, I drive quite a lot using a puller net, simply so that I do not have to sit there pulling on him. People can think what they like about driving with a puller net, but I think it is worse to sit there pulling hard on the horse’s mouth.*

*Bobbie: I think we have some bits that I do not like at all, such as the Crescendo bit, for example. But on the other hand, you could use a bit that is harsher, but never actually expose the horse to that harsher action, if you understand what I mean. In other words, a much sharper bit that you never have to use is better than a sharp bit that the horse does not respect. So, it is a bit unfortunate; we ought to have better rules regarding bits.*

The comments suggest that equipment choices were shaped by practical experience and beliefs about what works. Although the overall aim was similar—to achieve driveability without excessive force—participants differed in how they approached this. Elia questioned the effectiveness of sharper equipment, interpreting resistance as linked to fear or pain, whereas Robin and Bobbie described using additional tools or sharper bits to reduce the need for sustained or excessive rein tension. Although it is unclear what type of sharper bit Bobbie referred to, some sharper bits increase pressure within the horse’s mouth through leverage effects despite requiring less rein tension from the driver [1]. Consequently, lighter rein contact perceived by the driver does not necessarily mean that the horse experiences less oral pressure or discomfort.

Some participants discussed how competition regulations shaped equipment choices and perceived these regulations as sometimes limiting the possibility of using what they considered the most appropriate equipment for the horse. Bobbie, for example, questioned existing bit regulations in relation to how the severity of a bit is judged. These concerns were not limited to sharper bits; participants also mentioned milder alternatives, such as bitless noseband arrangements intended to reduce pressure on the mouth.

In addition to facilitating communication with the horse, participants described rein contact as providing information about the horse’s physical and mental state, as illustrated by Jessie:

*Jessie: It is sometimes difficult to know exactly what to expect from a horse that is new in training, but at least with young horses that you have had from the beginning, they are a certain way in the mouth from the start, so I think the warning bells should ring if they are suddenly not like that one day.*

By “warning bell”, Jessie referred to the possibility that the horse may be experiencing pain in the mouth or elsewhere in the body. Across the interviews, participants described changes in rein contact as practical warning signs indicating a possible underlying physical problem or discomfort. Changes such as increased or uneven rein tension were interpreted as potential signs of pain or discomfort, prompting further investigation. Participants commonly stated that the first response to driveability problems or altered rein tension should be to examine the horse’s mouth and body and seek veterinary assessment if necessary:

*Kay: Of course, you have to start with that, but we have said it a thousand times now […] It starts with having the veterinarian check why, for example, the horse is hanging on one rein. Or if its behaviour changes when you have previously been very satisfied with it, then the first thing you do is go to the veterinarian and have it checked. Then you check for lameness in the legs, and after that you check the oral cavity.*

Kay’s account illustrates that participants regarded changes in rein contact as cues for further investigation rather than problems to be addressed through equipment alone. Veterinary examination of the horse’s mouth and body was described as the appropriate first response. This interpretation is consistent with previous research suggesting that altered rein tension and resistance behaviours may indicate discomfort or pain [52,58] and with recommendations that recognising behavioural signs is central to identifying welfare problems [58].

Taken together, this theme highlights equipment as serving multiple functions in relation to driveability and performance. Participants described rein contact as facilitating communication with the horse while also providing information about its physical and mental state. Equipment choice was further described as involving ongoing negotiation between functionality, performance, and horse welfare.

## 4. General Discussion

This study explored how Swedish harness trainers and drivers conceptualise equine driveability and how they relate it to performance, welfare, equipment use, and rein tension in training and racing contexts. Across the three themes, driveability appeared relational and context-dependent.

### 4.1. Driveability as an Emergent and Relational Construct

Participants consistently defined a driveable horse as calm yet attentive and responsive to the driver’s signals; however, calmness was not framed as a passive or low-energy state. Rather, this description points towards regulated arousal: the ability to remain attentive and responsive under varying demands, including competitive pressure. Calm training was described as central to developing and maintaining this capacity, although participants also recognised limits to what training could achieve, particularly in relation to temperament and situational conditions. Equipment further shaped the interaction, influencing communication while introducing welfare considerations. These findings suggest that driveability cannot be reduced to temperament, arousal, responsiveness, or driver control alone. Instead, it may be understood as an emergent property of the driver–horse interaction, shaped by training practices and history, individual horse characteristics, equipment, and situational conditions.

Concepts such as rideability have sometimes been criticised for their apparent subjectivity and reliance on human judgement [15,19]. However, understanding driveability as relational also has implications for how it is assessed. The driver’s situated experience and professional judgement are therefore integral to both how driveability is enacted and how it is assessed in practice. The subjective nature of such assessments may therefore reflect the relational character of driveability, rather than simply representing a limitation. This interpretation may help explain why attempts to relate rideability or driveability to objective indicators have produced inconsistent findings [15,19,20].

### 4.2. Welfare and Performance: Alignment Rather than Opposition?

Within horse sport, welfare and performance are often framed as potentially competing priorities [1,49,59], particularly when competitive demands shape training and equipment choices. Participants acknowledged these tensions, including the demands and stresses of competition, but also described practices such as gradual training progression, management of stress and fatigue, and thoughtful equipment use as supporting driveability and performance while seeking to minimise discomfort and long-term harm. These accounts suggest that welfare and performance may, under certain relational and situational conditions, be mutually supportive rather than structurally opposed.

In this sense, driveability may function as a bridging construct. As a practitioner-recognised concept encompassing calmness, responsiveness, and communication, driveability provides a way of examining potential connections between welfare-relevant processes and performance. This does not imply that good performance demonstrates good welfare, or that welfare and performance necessarily align. Rather, the concept directs attention towards the conditions under which practices may support both. This interpretation is consistent with perspectives from ecological dynamics, which conceptualise performance as emerging through the interaction between the individual and its environment [60]. It also resonates with descriptions of equestrian performance as co-produced through ongoing adjustment within the human–horse partnership [47].

### 4.3. Equipment, Rein Contact, and Professional Judgement

Equipment formed an important part of the interaction through which driveability was experienced and managed. Rein tension was framed as both a means of regulation and a source of information through which drivers assessed the horse’s physical and mental state. Previous research has often measured and discussed rein tension as a potential welfare risk, particularly in relation to force levels, oral lesions, and conflict behaviours (e.g., [5,52]). The present findings add a complementary perspective by showing how practitioners understood rein contact. This is particularly important in the context of driving, where rein contact plays a particularly prominent role in communication between horse and driver.

Because rein contact involves both communication and the application of rein tension, equipment choices become ethically and practically important. Participants described an ongoing negotiation between comfort and functionality, sometimes viewing these as aligned and sometimes acknowledging trade-offs, particularly in competitive contexts. Equipment choices thus involved professional judgement about competing demands rather than straightforward technical decisions. This interpretation aligns with arguments that sustainable equestrian practice requires ongoing ethical reflection on how training and equipment choices affect horse welfare [51,56].

### 4.4. Implications for Future Research

Further research specific to harness racing is needed, given the emphasis on riding disciplines and thoroughbred racing in much of the existing scientific literature. Comparative studies across disciplines may help clarify which aspects of driveability are shared across equestrian sports and which are shaped by discipline-specific equipment, rules, and interactional norms. Observational studies of training and racing could complement interview findings by examining how drivers adjust rein contact and respond to changes in horses’ behaviour and arousal in practice.

Future research could also examine how practitioners’ assessments of driveability relate to measurable aspects of driver–horse interaction, combining qualitative accounts with measures of rein tension, behaviour, and locomotor asymmetry. While such integrative work has begun to emerge within the driving context [20], further studies could help to clarify how practitioners’ judgements correspond to behavioural and biomechanical indicators, and where they may diverge.

Finally, longitudinal research could investigate how training practices, rein tension, and equipment use develop within driver–horse partnerships and relate to welfare and performance over time. Such work would be particularly valuable for examining the conditions under which welfare-supportive practices and performance outcomes align or come into conflict.

### 4.5. Implications for Practice

Efforts to enhance driveability may benefit from considering how training practices, individual horse characteristics, and situational conditions interact. Participants consistently described practices such as gradual progression, attention to signs of fatigue and behavioural changes, and the creation of calm training environments. Recognising the potential contributions of these practices to driveability, horse welfare, and performance may help to inform training decisions.

Participants also described rein contact as a source of information about the horse, including changes that could prompt further examination or veterinary assessment. At the same time, participants acknowledged that excessive rein tension, mouth injuries, and practices that may compromise welfare do occur within the sport. The documented prevalence of oral lesions in trotters underscores the continued importance of monitoring oral health alongside changes in rein contact [3,4]. Professional dialogue, mentoring, and continuing education may provide opportunities to examine how rein tension is interpreted and managed in practice.

Notably, while participants described how they monitored and interpreted rein contact, they dedicated comparatively little explicit attention to how horses are taught to respond to rein cues. This does not necessarily mean that learning principles were absent from participants’ practice. Making these principles more explicit, including the timing and consistency of pressure–release signals [14,61], could support reflection on signalling practices and the sharing of practice-based knowledge.

At a structural level, competition regulations, professional education, and licensing systems also influence the conditions under which driveability is developed and expressed. Recognising their influence on training and racing practices may help to inform efforts to support both horse welfare and performance.

### 4.6. Methodological Reflections

This study was conducted within a critical realist framework and employed reflexive thematic analysis. These approaches were considered appropriate because the study aimed to explore how experienced trainers and drivers conceptualise driveability while recognising that such conceptualisations are shaped by practice and context. Reflexive thematic analysis [30] supported an interpretive analysis of participants’ accounts, allowing both explicit descriptions and underlying patterns of meaning to be explored. Critical realism [32,33] provided a framework for understanding participants’ accounts as situated interpretations of a real-world phenomenon, shaped by participants’ practical experience and context.

Consistent with reflexive thematic analysis, the themes presented in this study reflect the researchers’ interpretive engagement with the data and were shaped by the study’s theoretical perspective, research questions, and analytic decisions. Rather than seeking to eliminate researcher subjectivity, reflexive thematic analysis recognises the researchers’ interpretive role in knowledge production and treats reflexivity as an integral part of the analytic process. The themes should therefore not be understood as exhaustive or definitive representations of driveability, but as one interpretation of how experienced Swedish trainers and drivers conceptualised the phenomenon. Accordingly, the interpretation of driveability as relational and context-dependent should be understood as one way of making sense of the participants’ accounts rather than the only possible interpretation. Other theoretical perspectives might emphasise different aspects of driveability, such as individual traits or performance outcomes.

The researchers’ disciplinary backgrounds in equine science and welfare research, as well as familiarity with horse sport practice, informed both the interview questions and the analytical lens applied during analysis. Familiarity with debates surrounding horse welfare, rein tension, and equitation science may have sensitised the analysis to communication, welfare, and relational aspects of driveability. At the same time, efforts were made to remain attentive to participants’ own framings and terminology, allowing their conceptualisations to guide theme development. The interpretation of driveability as relational and context-dependent emerged through this iterative engagement with the data rather than being specified in advance, although the study’s theoretical perspective provided a framework for exploring such complexity.

The interviews were conducted with Swedish harness trainers and drivers who volunteered to participate. As such, the findings reflect the perspectives of practitioners willing to discuss their professional experiences in a research setting. As with all interview-based research, participants’ accounts may also have been shaped by the interview context, particularly when discussing welfare-sensitive issues such as rein tension and equipment use. The analytic focus, however, was not to verify practices, but to understand how participants make sense of driveability within their professional context.

In qualitative research, the aim is not statistical generalisation but to generate analytically transferable insights that may be relevant beyond the immediate study context [62]. The transferability of the findings depends on the extent to which readers judge them to be relevant in other contexts. To support such assessment, the study provides detailed descriptions of the research context, analytic process, and results. Care was also taken to describe participant characteristics in sufficient detail to support interpretation while preserving participant confidentiality.

## 5. Conclusions

This study suggests that Swedish harness trainers and drivers conceptualised driveability as relational, context-dependent, and not always fully controllable. Rather than reflecting a fixed characteristic of the horse, driveability was understood to emerge through the interaction between driver and horse. A driveable horse was described as calm, attentive, and responsive. Across three themes, participants described driveability as being shaped by training practices, individual horse characteristics, situational conditions, and equipment use.

These findings have significance for both research and practice. For research, they suggest that driveability is best studied as a relational and context-dependent concept, integrating practitioners’ assessments with objective measures rather than treating them as competing approaches. For practice, the findings highlight the importance of training approaches that support calmness and responsiveness, attentiveness to changes in rein contact as possible signs of discomfort, pain or altered mental state, and equipment decisions that consider both functionality and horse welfare. Understanding driveability in this way may help trainers, drivers, researchers, and the wider harness racing industry reflect on how performance and horse welfare can be supported together in everyday training and racing practice.

## Figures and Tables

**Table 1:** Overview of themes developed through reflexive thematic analysis of interviews with Swedish harness racing drivers and trainers.

Theme	Description
Calm training is key	Calm, gradual, low-intensity training was described as supporting driveability and performance by helping horses remain calm and responsive, while also helping trainers manage welfare challenges associated with racing.
One cannot always influence driveability	Driveability was described as shaped by inherited temperament, movement patterns, and situational conditions that could limit the driver’s ability to influence it directly, leading participants to adopt alternative driving strategies, such as driving behind another horse.
Equipment is a tool	Equipment, particularly the bit and rein contact, was described as a tool for communication, control, performance, and assessment of the horse’s physical and mental state. Its use involved balancing functionality with horse welfare.

## Data Availability

The interview data presented in this study are not publicly available to protect the confidentiality of the eight participating trainers and drivers, who may be identifiable from their responses.
